# Poor Appetite and Survival in Patients Admitted to an Acute Palliative Care Unit for Comprehensive Palliative Care

**DOI:** 10.3390/nu17111882

**Published:** 2025-05-30

**Authors:** Sebastiano Mercadante, Daniele Napolitano, Alessio Lo Cascio, Stefano Mancin, Alessandra Casuccio

**Affiliations:** 1 Main Regional of Supportive/Palliative Care, La Maddalena Cancer Center, 90146 Palermo, Italy; 2 CEMAD, Fondazione Policlinico Universitario Agostino Gemelli IRCCS, 00168 Roma, Italy; daniele.napolitano@policlinicogemelli.it; 3 Direction of Health Professions, La Maddalena Cancer Center, 90146 Palermo, Italy; 4 IRCCS Humanitas Research Hospital, Via Manzoni 56, Rozzano, 20089 Milan, Italy; stefano.mancin@humanitas.it; 5 Department of Health Promotion, Maternal and Infant Care, Internal Medicine and Medical Specialties, University of Palermo, 90127 Palermo, Italy; alessandra.casuccio@unipa.it

**Keywords:** advanced cancer, poor appetite, palliative care, acute palliative care unit, survival

## Abstract

Background/Objectives: Loss of appetite is a common symptom in patients with advanced cancer, and may contribute to patient deterioration. There is a lack of information about this issue, particularly in patients with advanced cancer admitted to an acute palliative care unit. The aims of this study were to assess appetite loss in patients admitted to an APCU and to investigate whether changes following comprehensive palliative care treatment are associated with survival. Materials and Methods: A consecutive sample of 520 patients admitted to the APCU was assessed. Patient characteristics and Edmonton Symptom Assessment Scale (ESAS) were measured at admission (T0) and after one week of comprehensive palliative care treatment (T7). Results: Of 381 patients screened, 208 (54.6%) had a poor appetite rating (≥4/10). Following comprehensive palliative care (T7), the number of patients with poor appetite significantly decreased to 116 (30%) (*p* < 0.0005). A multivariate regression analysis revealed that nausea (*p* = 0.002), weakness (*p* = 0.006), poor well-being (*p* = 0.017), and total ESAS score were correlated with poor appetite at T0. At T7, pain (*p* = 0.018), anxiety (*p* = 0.001), depression (*p* = 0.014), poor sleep (*p* = 0.047), drowsiness (*p* = 0.035), nausea (*p* = 0.018), weakness (*p* < 0.0005), poor well-being (*p* < 0.0005), and total ESAS score (*p* < 0.0005) were correlated with poor appetite. Survival was associated with a low Karnofsky (OR = 3.217(1.310–5.124), *p* = 0.001) and the presence of poor appetite at T7 (OR = −7.772(−14.662–−882), *p* = 0.027). Conclusions: A large proportion of patients admitted to an APCU present moderate-to-severe poor appetite. Clinical improvement of poor appetite is associated with improved survival.

## 1. Introduction

Poor appetite or loss of appetite, commonly described as a reduced or absent desire to eat, is among the most frequent and distressing symptoms experienced by patients with advanced illnesses, especially cancer and other severe chronic diseases [1,2]. Often termed anorexia, this symptom profoundly affects patients’ quality of life, nutritional status, and overall health outcomes, making it a significant clinical concern in palliative care settings [3,4]. Appetite loss encompasses various contributing factors, including physiological mechanisms, psychological distress, economic burdens, and social influences [5].

The regulation of appetite involves a highly sophisticated physiological system, comprising afferent signals that detect nutritional needs and efferent effectors that initiate eating behaviour [6]. Hunger stimulates food intake, and the ingestion of food triggers the release of satiety hormones, promoting digestion and a sense of fullness [3,4]. Under normal conditions, these mechanisms ensure energy balance and nutritional adequacy [7]. However, in patients with advanced cancer, this delicate balance often becomes disrupted, leading to substantial appetite loss and subsequent nutritional challenges [8,9,10].

The prevalence rates of anorexia among cancer patients vary significantly, ranging from approximately 30% to as high as 80%. Such variation largely results from differences in the patient populations studied, cancer types, disease stages, treatment regimens, and assessment methodologies [8,9]. This variability highlights the complexity and multifaceted nature of appetite disturbances, underscoring the need for targeted and individualized approaches to symptom management [11]. Notably, loss of appetite has severe implications, frequently leading to malnutrition, weight loss, decreased physical strength, impaired psychosocial well-being, and diminished functional independence [10,12]. Consequently, patients often experience heightened susceptibility to infections, increased fatigue, and reduced ability to perform daily activities, significantly decreasing their overall quality of life (Qol) [13,14,15].

Several physiological factors contribute intricately to appetite loss in the palliative care setting. Tumor progression commonly causes gastrointestinal disturbances, such as nausea, vomiting, early satiety, constipation, dysphagia, and reduced gastrointestinal motility, all of which directly limit food intake and worsen nutritional deficiencies [2,16]. Additionally, metabolic alterations induced by tumors involve systemic inflammation and disrupted energy metabolism, which play crucial roles [17]. These changes often manifest as cancer cachexia, a severe metabolic syndrome characterized by significant weight loss, primarily affecting skeletal muscle mass, and resistant to standard nutritional interventions [18,19].

Beyond the physiological mechanisms, changes in taste and smell, gastrointestinal motility issues, nausea, and constipation significantly influence appetite. Pain, emotional distress, anxiety, depression, and existential concerns frequently worsen anorexia, highlighting the importance of a comprehensive, multidisciplinary approach to care [16,18]. These symptoms may arise as the side effects of cancer treatments or directly from tumor progression, necessitating holistic interventions that integrate medical, nutritional, psychological, and social support systems [6,12,19,20,21].

Psychological factors add further complexity to the management of anorexia in advanced cancer patients. Anxiety, depression, and emotional distress often coexist with appetite reduction, significantly increasing the symptom burden [22]. Effective management requires multidimensional palliative strategies that include psychological assessment, counseling, emotional support, and standard medical care [6,12]. Supportive social environments have demonstrated notable benefits in mitigating appetite disturbances, emphasizing the importance of patient-centered care strategies [23,24].

The assessment of appetite loss presents significant challenges due to its inherently subjective nature. Currently, no reliable biomarkers exist to objectively quantify anorexia, making patient-reported outcome measures essential for symptom evaluation [20,21,25]. Among these, the Edmonton Symptom Assessment Scale (ESAS) effectively captures patient-reported severity of anorexia using a numerical rating scale, providing valuable insights for clinical decision-making [26,27]. Alternative assessment tools, such as visual analogue scales and specialized nutritional questionnaires, offer additional options, though they are less frequently used [28].

Advancements in cancer therapies have increased the necessity for Acute Palliative Care Unit (APCU) admissions, even in advanced disease stages, to manage intensified symptoms through multidisciplinary supportive care [29]. Specialized acute palliative care units focus on comprehensive interventions aimed at relieving complex symptoms, improving comfort, and enhancing quality of life [30,31]. Despite the high prevalence and significant impact of anorexia, current research notably lacks robust evidence specifically addressing this symptom in APCU settings. This lack of data underscores a critical knowledge gap regarding the trajectory of anorexia, the effectiveness of multifaceted interventions, and their implications for patient outcomes, particularly survival [32].

Given this clear gap in the literature and the multifaceted nature of appetite loss, rigorous and systematic research is essential. Emerging studies suggest potential associations between improvements in appetite following structured interventions and better survival outcomes. However, these associations remain underexplored, highlighting the urgent need for dedicated research to clarify the prognostic significance of anorexia within APCU settings [30,33]. Understanding these relationships could substantially improve clinical decision-making, patient management, and overall palliative care quality.

Therapeutic strategies for managing anorexia in palliative care include pharmacological treatments such as corticosteroids, progestogens, and emerging agents like anmorelin, which are designed to enhance appetite and improve nutritional outcomes [31].

Non-pharmacological interventions, such as tailored nutritional counseling, dietary adjustments, psychotherapy, and targeted management of symptoms like nausea, pain, and fatigue, also play crucial roles [34,35].

Therefore, given the limitations and gaps identified in the current literature, the primary aim of this study was to assess appetite loss in patients at admission and at discharge after receiving comprehensive supportive treatment in an APCU. The second outcome was to assess whether changes after comprehensive palliative care treatment may be associated with survival.

## 2. Materials and Methods

This study represents a prospectively planned secondary analysis of a prospectively maintained clinical database, designed to capture the real-world clinical trajectory of patients treated in an APCU. The study was approved by the local Ethical Committee (Ethical Committee Palermo 1, identification number PSCU0005/2023 of 14 November 2023). Patient informed consent was obtained. Over a continuous 14-month enrollment period, all of the 520 admissions to the APCU were recorded in the database; no patient was omitted, minimizing the risk of selection bias and ensuring that the study cohort reflects routine clinical practice. For the current analysis, two additional eligibility criteria were applied. First, comprehensive follow-up data on vital status had to be available, obtained through structured telephone interviews conducted by dedicated research nurses within the two months following the end of the recruitment phase. Second, a complete Edmonton Symptom Assessment System (ESAS) questionnaire must have been successfully administered at the prespecified time points. Only patients who were mentally competent to give informed consent and physically and cognitively able to understand and score each ESAS item were included in the analysis cohort. Conversely, individuals were excluded if, in the admitting physician’s judgement, they appeared to be approaching imminent death, had severe cognitive impairment denoted by a Memorial Delirium Assessment Scale score ≥ 13, or died before the first post-admission assessment could be performed. This rigorous, yet practical, set of inclusion and exclusion criteria produced a clinically homogeneous cohort that is representative of day-to-day APCU activity [35]. Patients who died during admission were excluded.

### 2.1. Measurements

The characteristics of patients, including age, gender, cancer diagnosis, and ongoing anticancer treatment, were collected. ESAS was measured at admission (T0) and after one week of comprehensive palliative care treatment (T7). ESAS is a self-reported tool measuring the intensity of physical and psychological symptoms and is sensitive to changes reported by patients after a therapeutic intervention. A numeric rating scale for each symptom from 0 (no symptom) to 10 (worst intensity) over the past 24 h is used for all ESAS items [27]. The ESAS was filled out by physicians who helped patients provide the data. Traditional ESAS items include pain, weakness, nausea, depression, anxiety, drowsiness, dyspnea, poor appetite, poor sleep, and poor well-being on a numeric rating scale ranging from 0 to 10 [36]. Among the ESAS items, poor appetite is measured on a scale from 0 (good appetite) to 10 (no appetite). All patients were assessed by a specialist palliative care team and received comprehensive palliative care treatment, which included proper assessment, recognition of possible reversible causes of symptoms, and symptom management on an individual basis according to the patient’s specific needs [37].

### 2.2. Statistics

Continuous data were presented as mean ± SD. Frequency distributions were analyzed using Pearson’s chi-square test and Fisher’s exact test, as appropriate. The paired Wilcoxon signed-rank test was used to compare pain and other symptom scores over the specified time intervals. The correlation between various clinical parameters (independent variables) and patient groups with different levels of poor appetite (≥4/10 vs. <4/10) (dependent variable) was evaluated using univariate (Crude OR) and multivariate regression models (Adjusted OR), with 95% confidence intervals reported. A sample size of 54 patients per group (with or without poor appetite, ≥4/10 vs. <4/10) provided 80% power with an alpha (α) of 0.05, enabling the detection of a 30% difference in symptom score reduction between groups, assuming a standard deviation (SD) of ±3. The sample size calculation aimed at 95% power with α = 0.05.

## 3. Results

Of 520 patients admitted to the APCU in the period under consideration, 381 patients were screened. Sixty patients died in the unit, 77 had incomplete data, and 2 patients had severe cognitive disorders. The characteristics of the patients are listed in Table 1.

Among the remaining 381 patients, 208 (54.6%) had a poor appetite intensity of ≥4/10, with 103 patients (27%) having ≥7/10 on ESAS at T0. After a comprehensive palliative care treatment (T7), the number of patients with poor appetite and severe poor appetite significantly decreased: 116 (30%) and 35 (9.1%) at T0 and T7, respectively (*p* < 0.0005). Data regarding the changes in the ESAS items from T0 to T7 are reported in Table 2. Changes in symptom intensity were highly significant for all items.

In the univariate analysis, age (*p* = 0.026), anxiety (*p* = 0.005), depression (*p* = 0.004), nausea (*p* < 0.0005), weakness (*p* < 0.0005), poor well-being (*p* < 0.0005), and total ESAS (*p* <0.0005) were significantly correlated with poor appetite at T0. At T7, the level of appetite was related to Karnofsky (*p* = 0.046), pain (*p* = 0.003), anxiety (*p* < 0.0005), depression (*p* < 0.0005), poor sleep (*p* < 0.0005), drowsiness (*p* < 0.0005), nausea (*p* = 0.001), weakness (*p* < 0.0005), poor well-being (*p* < 0.0005), and total ESAS (*p* < 0.0005). At the multivariate regression analysis, nausea (*p* = 0.002), weakness (*p* = 0.006), poor well-being (*p* = 0.017), and total ESAS were correlated with poor appetite at T0 (Table 3)

At T7, pain (*p* = 0.018), anxiety (*p* = 0.001), depression *(p* = 0.014), poor sleep (*p* = 0.047), drowsiness (*p* = 0.035), nausea (*p* = 0.018), weakness (*p* < 0.0005), poor well-being (*p* < 0.0005), and total ESAS (*p* < 0.0005) were correlated with poor appetite (Table 4).

Among the 208 patients presenting with moderate-to-severe appetite loss at baseline (T0; ESAS score ≥ 4/10), we examined the symptom evolution after one week of comprehensive palliative care (T7) and its impact on survival. Of these, 124 patients (59.6%) improved to a score <4 at T7 and were considered responders. The remaining 84 patients (40.4%) still reported poor appetite (≥4/10), including 58 patients (27.9%) with persistent severe loss (≥7/10). The transition in appetite categories between T0 and T7 is shown in Table 5. A statistically significant shift towards lower symptom intensity was observed (*p* < 0.0005, McNemar test).

Regarding overall survival, no differences were observed between patients with moderate–severe poor appetite (≥4/10) and mild poor appetite (<4/10) at T0 (mean 74 ± 45 days versus 77 ± 135). days. However, at T7, survival was significantly shorter (*p* = 0.001) in patients who had poor appetite ≥4/10 (mean 31 ± 45 days) than in patients with mild poor appetite (<4/10) (mean 95 ± 161 days). Survival was associated with a low Karnofsky (OR = 3.217(1.310–5.124), *p* = 0.001) and poor appetite at T7 (OR = −7.772(−14.662–−882), *p* = 0.027).

## 4. Discussion

This study demonstrated that a significant proportion of patients admitted to an APCU present with moderate-to-severe poor appetite. This condition was independently associated with the level of Karnofsky Performance Status, pain, anxiety, depression, poor sleep, drowsiness, nausea, weakness, poor well-being, and total ESAS scores at T0, as well as at T7 after one week of comprehensive palliative care [38]. Of the 381 evaluable patients, 54% had an Edmonton Symptom Assessment System (ESAS) score ≥ 4/10, and just over one in four reached the extreme threshold of ≥7/10. These proportions are at the upper end of the 30–60% prevalence range reported in previous multicenter surveys, highlighting how profoundly the symptom influences patients’ experience of advanced disease. Given that the ESAS captures only the preceding 24 h, these figures likely underestimate the cumulative impact of appetite loss throughout the terminal illness trajectory [5]. These symptoms improved collectively, maintaining the same pattern of association among them. The second key finding relates to survival. While no differences were observed at T0 between patients with different levels of poor appetite, patients who remained poorly responsive to treatment at T7, maintaining high levels of poor appetite, had a shorter survival compared to those who showed improvement. Thus, intensive treatment in the APCU may have a positive influence on survival. Previous studies have indicated that reduced food intake, loss of appetite, and inflammation (measured as elevated C-reactive protein levels) are risk factors for overall survival in cancer patients [4,39,40,41]. In a post hoc analysis, moderate-to-poor appetite was significantly linked to higher mortality compared to reporting a good appetite [9,42]. In a secondary analysis of a study investigating the provision of palliative care in the APCU, nearly two-thirds of admitted patients experienced moderate-to-severe appetite loss. Patients with appetite loss also reported more nausea, depression, fatigue, dyspnea, and anxiety. However, no significant changes in symptom intensity were observed, although an improvement in appetite was noted in about one third of patients, but this was not quantified and could coincide with an alleviation of fatigue [5]. However, these studies had a cross-sectional design, lacking reporting of specialist intervention, or focused on patients with limited life expectancy, referred to as specific palliative care. In our study, patients received specialist intervention in the APCU, with highly significant improvements in ESAS symptoms, including poor appetite. Comparable, but less detailed, observations have been reported in other cohorts. Helgesen and colleagues found that moderate-to-severe levels of poor appetite were present in 62% of Norwegian cancer admissions, with qualitative improvements in roughly two thirds during hospitalization, accompanied by decreases in nausea, depression, and fatigue [5]. However, the lack of post-discharge follow-up prevented an assessment of prognostic significance. Our study extends those initial findings by demonstrating that the persistence of poor appetite after a short, protocolized intervention period predicts significantly reduced survival. Juxtaposing this evidence with studies from non-malignant chronic diseases reveals both similarities and nuances. In acute decompensated heart failure, even a modest decline in appetite at discharge doubled one-year mortality, despite a baseline prevalence of only 12% [43]. Longitudinal studies in chronic heart failure cohorts show that poor appetite remains compromised in up to 40% at 18 months, and is linked to systemic congestion, inflammatory activation, and fatigue [44]. These data align with our findings, suggesting that appetite reflects overall physiological reserve, regardless of the underlying diagnosis.

In chronic obstructive pulmonary disease (COPD), meta-analyses indicate that 25–40% of patients develop protein–energy malnutrition driven by elevated energy expenditure, dyspnea during meals, and systemic inflammation; appetite loss is often the initial symptom and is independently associated with exacerbation frequency and mortality [45]. Although COPD generally progresses more slowly than advanced cancer, the mechanistic overlap, including cytokine-mediated anorexigenic signaling, altered gut-brain axis, and co-existing depression, supports the need for similar multi-disciplinary strategies.

End-stage renal disease provides an additional parallel: approximately 42% of hemodialysis recipients experience appetite impairment, which predicts protein energy wasting, hospitalization, and death [46]. Uraemic taste alterations and stringent dietary restrictions are disease-specific contributors, yet the central role of inflammation, psychological distress, and catabolic drive recapitulates the pattern observed in oncology. These findings support a unifying model in which poor appetite emerges when metabolic stress, inflammation, and psychosocial distress exceed the body’s homeostatic capacity. The pace and reversibility of the symptom, however, depend on the primary pathology: in cancer, the trajectory is highly dynamic, making poor appetite particularly amenable to rapid improvement if addressed promptly and comprehensively. Our findings indicate that improvement in loss of appetite was associated with increased survival compared to the baseline assessment. This novel observation has not been previously reported, underscoring the importance of an APCU, where intensive symptom management can lead to rapid improvements in overall condition.

A limitation of our study is that it was based on a single-center experience, which is dedicated exclusively to intensive treatment and research. Therefore, these findings may not be generalizable to other settings. Another limitation is the short follow-up period (two months) after the end of the recruitment phase, and the incomplete data available for overall survival. Two months also represent the mean survival of patients admitted to the APCU [46]. Additionally, due to the absence of a public registry, patients were contacted personally by phone, and some were lost to follow-up. The use of medications, including specific orexigenic drugs, and data on patients already using medications were not collected. This was because patients received personalized treatment within comprehensive management, rather than following a standardized protocol, which would be unreliable in a real-world setting like this. Finally, dying patients were excluded because it was not possible to properly assess them. A strength of our study is that participants were thoroughly assessed according to APCU policy and received personalized treatment tailored to individual needs to optimize outcomes.

## 5. Conclusions

In conclusion, this study’s data demonstrate that many patients admitted to an APCU present with moderate-to-severe poor appetite, which is associated with shorter survival. However, clinical improvement of the item poor appetite was linked to increased survival among patients who responded to comprehensive palliative care. Therefore, intensive treatment in the APCU may positively influence patient survival. Predicting survival time remains challenging in palliative cancer care, underscoring the importance of evaluating such factors dynamically to identify more reliable prognostic markers. Future research should aim to confirm these findings through collaborative studies involving APCU or other palliative care settings and further investigate the role of symptom clustering within the ESAS, which may influence the stability of multivariate models.

## Figures and Tables

**Table 1:** Characteristics of patients.

Variable	Value
Number of patients	381
Age, yrs (SD)	67.3 (±11.6)
Gender (M/F)	170/211
Cancer diagnosis	
Lung	117
Breast	50
Gastrointestinal	117
Urogenital	45
Head-neck	4
Others	23
unknown	25
On therapy	167
Uncertain	43
Off therapy	103
Naive	68
Poor appetite <4	173 (45%)
Poor appetite 4–6	105 (28%)
Poor appetite ≥7	103 (27%)
Survival, days (SD)	75 (140)

SD: Standard deviation; M: male; F: female.

**Table 2:** Changes in ESAS items after comprehensive palliative care treatment (from T0 to T7).

Variable	T0	T7	*p*
Pain	3.8 (2.8)	1.7 (1.8)	<0.0005
Dyspnea	1.2 (2.1)	0.5 (1.3)	<0.0005
Depression	2.3 (3.0)	1.1 (2.1)	<0.0005
Anxiety	2.9 (3.0)	1.4 (2.2)	<0.0005
weakness	5.6 (2.8)	3.4 (2.6)	<0.0005
Drowsiness	2.7 (2.6)	1.7 (2.1)	<0.0005
Poor sleep	3.8 (3.2)	1.8 (2.4)	<0.0005
Nausea	1.0 (2.1)	0.3 (1.1)	<0.0005
Poor appetite	3.9 (3.4)	2.1 (2.7)	<0.0005
Poor well-being	5.0 (2.8)	2.7 (2.5)	<0.0005
Total	32.0 (14.6)	16.8 (12.6)	<0.0005

*p*-values < 0.05 are considered statistically significant.

**Table 3:** Univariate (Crude OR) and multivariate (AdjOR) regression analysis for the correlation among the characteristics of patients and poor appetite at T0 (≥4/10 VS. <4/10).

	Poor Appetite at T0			
Variable	Crude OR (95% CI)	*p*-Value	Adj-OR (95% CI)	*p*-Value
Age	1.020 (1.002–1.039)	0.026	1.023(1.004–1.042)	0.020
Sex (F vs. M)	1.229 (0.819–1.844)	0.320	-	-
Karnofsky	0.986 (0.966–1.007)	0.186	-	-
On therapy at T0	1.068 (0.852–1.338)	0.570	-	-
Pain at T0	0.988 (0.920–1.061)	0.738	-	-
Dyspnea at T0	1.100 (0.998–1.212)	0.055	1.080 (0.976–1.195)	0.136
Anxiety at T0	1.101 (1.029–1.179)	0.005	1.047 (0.957–1.146)	0.314
Depression at T0	1.110 (1.035–1.191)	0.004	1.077 (0.983–1.180)	0.113
Poor sleep at T0	1.011 (0.951–1.076)	0.719	-	-
Drowsiness at T0	1.025 (0.949–1.107)	0.523	-	-
Nausea at T0	1.278 (1.133–1.443)	<0.0005	1.216 (1.073–1.377)	0.002
Weakness at T0	1.210 (1.121–1.307)	<0.0005	1.133 (1.036–1.238)	0.006
Poor well-being at T0	1.199 (1.112–1.294)	<0.0005	1.113 (1.019–1.216)	0.017
Total ESAS at T0	1.072 (1.053–1.092)	<0.0005	1.074 (1.054–1.095)	<0.0005

OR = odds ratio. Adj-OR = adjusted odds ratio. CI = confidence interval; M: male; F: female; ESAS (Edmonton Symptom Assessment System), *p*-values < 0.05 are considered statistically significant.

**Table 4:** Univariate (Crude OR) and multivariate (AdjOR) regression analysis for the correlation among the characteristics of patients and poor appetite at T7 (≥4/10 VS. <4/10).

	Poor Appetite at T7 (Yes vs. No)			
Variable	Crude OR (95% CI)	*p*-Value	Adj-OR (95% CI)	*p*-Value
Age	1.015 (0.996–1.035)	0.130	-	-
Sex (F vs. M)	0.894 (0.577–1.385)	0.616	-	-
Karnofsky	0.977 (0.956–0.999)	0.046	0.979 (0.956–1.001)	0.061
In therapy at T7	1.366 (0.949–1.967)	0.094	-	-
Pain at T7	1.201 (1.065–1.354)	0.003	1.165 (1.027–1.321)	0.018
Dyspnea at T7	1.121 (0.959–1.311)	0.151	-	-
Anxiety at T7	1.239 (1.125–1.365)	<0.0005	1.197 (1.076–1.330)	0.001
Depression at T7	1.267 (1.146–1.400)	<0.0005	1.187 (1.036–1.362)	0.014
Poor sleep at T7	1.187 (1.088–1.294)	<0.0005	1.1071.001–1.225)	0.047
Drowsiness at T7	1.245 (1.122–1.381)	<0.0005	1.134 (1.009–1.274)	0.035
Nausea at T7	1.402 (1.150–1.710)	0.001	1.294 (1.046–1.601)	0.018
Weakness at T7	1.607 (1.436–1.798)	<0.0005	1.538 (1.356–1.745)	<0.0005
Poor well-being at T7	1.507 (1.357–1.673)	<0.0005	1.404 (1.246–1.583)	<0.0005
Total ESAS at T7	1.151 (1.117–1.186)	<0.0005	1.445 (1.335–1.564)	<0.0005

OR = odds ratio. Adj-OR = adjusted odds ratio. CI = confidence interval; M: male; F: female; ESAS (Edmonton Symptom Assessment System). *p*-values < 0.05 are considered statistically significant.

**Table 5:** Appetite score transitions from baseline (T0) to one week (T7).

	T7				
	Poor Appetite <4	Poor Appetite 4–6	Poor Appetite ≥7	Total	*p*
Poor appetite <4	141	25	7	173	<0.0005 *
Poor appetite 4–6	66	31	8	105	
Poor appetite ≥7	58	25	20	103	
Total	265	81	35	381	

* are considered statistically significant (McNemar test).

## Data Availability

The original contributions presented in this study are included in the article. Further inquiries can be directed to the corresponding authors.
